# Transforming Growth Factor-Beta and Sonic Hedgehog Signaling in Palatal Epithelium Regulate Tenascin-C Expression in Palatal Mesenchyme During Soft Palate Development

**DOI:** 10.3389/fphys.2020.00532

**Published:** 2020-06-04

**Authors:** Shirabe Ohki, Kyoko Oka, Kayoko Ogata, Shigeru Okuhara, Mihoko Rikitake, Masako Toda-Nakamura, Shougo Tamura, Masao Ozaki, Sachiko Iseki, Takayoshi Sakai

**Affiliations:** ^1^Section of Pediatric Dentistry, Department of Oral Growth and Development, Fukuoka Dental College, Fukuoka, Japan; ^2^Oral Medicine Research Center, Fukuoka Dental College, Fukuoka, Japan; ^3^Section of Functional Structure, Department of Morphological Biology, Fukuoka Dental College, Fukuoka, Japan; ^4^Section of Molecular Craniofacial Embryology, Graduate School of Dental and Medical Sciences, Tokyo Medical and Dental University, Tokyo, Japan; ^5^Department of Oral-Facial Disorders, Osaka University Graduate School of Dentistry, Osaka, Japan

**Keywords:** soft palate, palatogenesis, tumor growth factor-beta, tenascin-C, sonic hedgehog

## Abstract

During palatogenesis, the palatal shelves first grow vertically on either side of the tongue before changing their direction of growth to horizontal. The extracellular matrix (ECM) plays an important role in these dynamic changes in palatal shelf morphology. Tenascin-C (TNC) is an ECM glycoprotein that shows unique expression in the posterior part of the palatal shelf, but little is known about the regulation of TNC expression. Since transforming growth factor-beta-3 (TGF-β3) and sonic hedgehog (SHH) signaling are known to play important roles in palatogenesis, we investigated whether TGF-β3 and SHH are involved in the regulation of TNC expression in the developing palate. TGF-β3 increased the expression of TNC mRNA and protein in primary mouse embryonic palatal mesenchymal cells (MEPM) obtained from palatal mesenchyme dissected at embryonic day 13.5–14.0. Interestingly, immunohistochemistry experiments revealed that TNC expression was diminished in *K14-cre*;*Tgfbr2*^*fl/fl*^ mice that lack the TGF-β type II receptor in palatal epithelial cells and exhibit cleft soft palate, whereas TNC expression was maintained in *Wnt1-cre*;*Tgfbr2*^*fl/fl*^ mice that lack the TGF-β type II receptor in palatal mesenchymal cells and exhibit a complete cleft palate. SHH also increased the expression of TNC mRNA and protein in MEPM cells. However, although TGF-β3 up-regulated TNC mRNA and protein expression in O9-1 cells (a cranial neural crest cell line), SHH did not. Furthermore, TGF-β inhibited the expression of osteoblastic differentiation markers (osterix and alkaline phosphatase) and induced the expression of fibroblastic markers (fibronectin and periostin) in O9-1 cells, whereas SHH did not affect the expression of osteoblastic and fibroblastic markers in O9-1 cells. However, immunohistochemistry experiments showed that TNC expression was diminished in the posterior palatal shelves of *Shh^–/+^*;*MFCS4*^+/–^ mice, which have deficient SHH signaling in the posterior palatal epithelium. Taken together, our findings support the proposal that TGF-β and SHH signaling in palatal epithelium co-ordinate the expression of TNC in the posterior palatal mesenchyme through a paracrine mechanism. This signal cascade may work in the later stage of palatogenesis when cranial neural crest cells have differentiated into fibroblast-like cells. The spatiotemporal regulation of ECM-related proteins by TGF-β and SHH signaling may contribute not only to tissue construction but also to cell differentiation or determination along the anterior–posterior axis of the palatal shelves.

## Introduction

Cleft palate is one of the most common craniofacial birth defects in humans. Multidisciplinary research into palatal development has yielded important insights into the mechanisms underlying cleft palate (Li et al., 2017). However, the causes of partial clefts, especially cleft soft palate and submucous cleft palate, remain incompletely understood.

The palatal shelf starts to form on embryonic day 11.5 (E11.5). The mesenchymal tissue of the palatal shelf is mostly composed of cranial neural crest-derived cells that are divided into distinct regions along their anterior–posterior axis (Chai et al., 2000; Oka et al., 2012; Smith et al., 2012). The cranial neural crest-derived mesenchymal cells give rise to the bony hard palate in the anterior and middle parts and to the connective tissue of the soft palate in the posterior part. Previously we reported that transforming growth factor-beta (TGF-β) induces the co-expression of type I collagen and periostin in the region of the posterior palatal mesenchyme that forms the palatine aponeurosis, an important structure for palatine muscle formation (Oka et al., 2012). TGF-β signaling is widely recognized as playing an important role in the synthesis, deposition and regulation of the extracellular matrix (ECM) (Yun et al., 2019). During palatogenesis, the pattern of ECM expression along the anterior–posterior axis may regulate and support osteoblastic or fibroblastic differentiation of cranial neural crest-derived mesenchymal cells.

Numerous growth factors, including TGF-β, have been implicated in the development of cleft palate (Li et al., 2017). TGF-β ligands and receptors are ubiquitously expressed in palatal epithelium and mesenchyme, but their roles differ between the epithelium and mesenchyme along the anterior–posterior axis (Iwata et al., 2011). Furthermore, mutations of individual TGF-β ligands and receptors in mice lead to distinctive types of cleft palate caused by different mechanisms. For example, it has been reported that TGF-β signaling regulates cell proliferation in palatal mesenchyme and that loss of the TGF-β type II receptor in palatal mesenchyme leads to a complete cleft palate (Ito et al., 2003). On the other hand, TGF-β signaling in palatal epithelium regulates palatal adhesion and epithelial seam degradation for fusion of the palatal shelves. TGF-β3 is characteristically expressed in the medial edge epithelium located at the adhesive region of each palatal shelf (Taya et al., 1999). Interestingly, loss of the TGF-β type I and II receptors in palatal epithelium leads to a partial cleft in the posterior region (Dudas et al., 2006; Xu et al., 2006). This latter finding raises the possibility that TGF-β signaling in palatal epithelium is involved not only in epithelial seam degradation but also in the formation of the posterior palatal region. Therefore, we have been interested in exploring the influence of palatal TGF-β signaling on ECM expression patterns during epithelial–mesenchymal interactions.

The sonic hedgehog (SHH) signaling pathway plays a crucial role in embryogenesis and is involved in epithelial–mesenchymal interactions (Bitgood and McMahon, 1995). SHH is also thought to be important for palatal development. For example, SHH was shown to regulate palatal shelf morphogenesis (Rice et al., 2006). Furthermore, SHH signaling was found to modulate palatal growth through epithelial–mesenchymal interactions, whereby alterations in the expressions of transcription factors in the palatal mesenchyme influenced the proliferation of palatal epithelial cells (Lan and Jiang, 2009). Notably, SHH has been implicated in the regulation of palatal fusion by TGF-β3 (Sasaki et al., 2007), and abnormal SHH signaling in mice is associated with cleft palate (Hammond et al., 2018; Li et al., 2018).

Tenascins are a family of large oligomeric ECM glycoproteins comprising four members in vertebrates (-C, -R, -X, and -W). All tenascin subunits are composed of an N-terminal domain, a variable number of tandem epidermal growth factor and fibronectin type III repeats, and a fibrinogen homology domain at the C-terminus (Chiquet-Ehrismann and Chiquet, 2003; Chiquet-Ehrismann and Tucker, 2011). Among the tenascin family, tenascin-C (TNC) is widely expressed in mesenchymal tissue at sites of epithelial–mesenchymal interactions and around motile cells, including neural crest cells and migrating neuroblasts and glial precursors (Chiquet-Ehrismann et al., 2014). It has been reported that TNC is expressed in palatal mesenchyme during palatogenesis (Chiquet et al., 2016; Li et al., 2017; Paiva et al., 2019). However, the mechanisms regulating this characteristic expression of TNC during palatogenesis have not been elucidated.

Here, we show that TNC expression in the palatal mesenchyme is regulated by both TGF-β and SHH signaling in the palatal epithelium. However, the regulation of TNC expression by these signaling pathways differs and depends on the developmental stage of the palatal mesenchyme. Spatiotemporal regulation of TNC expression in the palatal mesenchyme by TGF-β and SHH in the palatal epithelium might be critical for the characteristic development of the hard and soft palate.

## Materials and Methods

### Animals

All procedures for animal care were reviewed and approved by the Animal Experiment Committee of Fukuoka Dental College, Fukuoka, Japan (nos. 17012 and 18005). ICR mice (Kyudo Co., Tosu, Japan) were used for immunohistochemical analyses and cell culture experiments. Male mice carrying the K14-Cre allele and Wnt1-Cre allele were crossed with *Tgfbr2*^*fl/fl*^ females to generate *K14-Cre*;*Tgfbr2*^*fl/fl*^ mice, which lack the TGF-β type II receptor in palatal epithelial cells, and *Wnt1-Cre*;*Tgfbr2*^*fl/fl*^ mice, which lack the TGF-β type II receptor in palatal mesenchymal cells (Chytil et al., 2002; Ito et al., 2003; Andl et al., 2004). In compound heterozygous *Shh^–/+^*;*MFCS4*^+/–^ mice (Amano et al., 2009; Sagai et al., 2009), the *MFCS4* long-distance Shh enhancer is deleted on one allele, whereas the *Shh* gene remains intact on the same allele, resulting in reduced levels of SHH. To achieve *Shh* deletion at specific developmental stages, *pCag-CreERTM*;*Shh*^*flox/+*^ mice were mated with *Shh*^*flox/+*^ mice (Dassule et al., 2000; Hayashi and McMahon, 2002), and pregnant mice were administered tamoxifen by intraperitoneal injection (75 mg/kg, equivalent to 3 mg per 40 g body weight) at the appropriate developmental stage. For the experiments, comparisons were made between each of the above mutants and wild-type littermates.

### Palatal Development Stages

We selected the developmental stages for analysis on the basis of previous reports (Oka et al., 2012; Higa et al., 2016). The palatal shelves are evident on either side of the tongue at E13.5 and are elevated above the tongue at around E14.0. Palatal mesenchymal cells undergo proliferation rather than differentiation during E13.5–14.0, and at this stage it is difficult to distinguish between the hard and soft palate. At E14.5, the bone matrix of the maxilla of the hard palate and the sphenoid bone of the soft palate (which represent characteristic components of each palate) are detectable in histological section. After E14.5, each of the neural crest cell-derived osteogenic and fibrogenic components of the hard and soft palates is clearly identifiable in histological section. The skeletal muscle cells that originate from mesoderm-derived cells do not exist in the palatal shelves of mice (Grimaldi et al., 2015). Thus, palatal mesenchymal cells before E15.5 are mainly derived from cranial neural crest cells.

### Immunohistochemistry

Immunohistochemistry was performed on palatal tissues obtained from mice at embryonic stages E13.5 to E15.5. The tissues were fixed with 10% neutral buffered formalin or 4% paraformaldehyde in phosphate-buffered saline, dehydrated in graded ethanol solutions, embedded in paraffin, and cut into 6-μm sections for immunostaining. The primary antibodies used were rat anti-TNC monoclonal antibody (1:100 dilution; MAB2138, R&D Systems, Minneapolis, MN, United States) and rabbit anti-cytokeratin broad-spectrum screening polyclonal antibody (1:200 dilution; Z0622, Dako Cytomation, Carpinteria, CA, United States), and these were applied for 1 h at room temperature. The secondary antibodies used were Alexa Fluor^®^ 488 anti-rabbit immunoglobulin G (IgG), Alexa Fluor^®^ 594 goat anti-rat IgG, and biotin-conjugated goat anti-rat IgG H&L (Abcam, Tokyo, Japan). The nuclei were counterstained with DAPI (4′,6-diamidino-2-phenylindole), which was present in the mounting medium (Vectashield, Vector Laboratories, Burlingame, CA, United States) or Methyl Green (Merck Millipore, Darmstadt, Hessen, Germany). Specimens treated with biotin-conjugated secondary antibody were sensitized using streptavidin peroxidase (Vector Laboratories) and visualized using a diaminobenzidine kit (Nichirei Biosciences Inc., Tokyo, Japan). The specimens were observed using a fluorescence microscope (BZ9000; Keyence, Osaka, Japan).

### Real-Time Polymerase Chain Reaction (PCR)

Palatal shelves were dissected from ICR mouse embryos at E13.5, E14.5, and E15.5 and divided into anterior and posterior portions. The position of the border between the anterior and posterior portions of the palatal shelf was defined with reference to a 3D reconstruction of the mouse embryo provided by NIH FaceBase^1^. Using morphologic observations, it was straightforward to distinguish the much smaller posterior portion of the palatal shelf from the anterior portion in the secondary palate. In addition, visualization of the developing rugae under a stereomicroscope from E14.5 also helped to define the border between the anterior and posterior portions. RNA was isolated from tissue using TRIzol reagent (Thermo Fisher Scientific, Tokyo, Japan) and extracted from cells using the RNeasy^®^ Mini kit (Qiagen, Valencia, CA, United States). cDNA was synthesized using PrimeScript^®^ II Reverse Transcriptase (Takara, Otsu, Japan). After mixing each cDNA with SsoAdvanced^TM^ Universal SYBR^®^ Green Supermix (Bio-Rad, Hercules, CA, United States), amplification was performed in a CFX96^TM^ Real-Time System (Bio-Rad). Primer sequences for the genes investigated are shown in Table 1. Results were standardized to the expression of the glyceraldehyde 3-phosphate dehydrogenase gene (*Gapdh*), and fold differences in the expression of each gene were calculated according to the ΔΔCT method with normalization to *Gapdh*.

**TABLE 1:** Primers used for real-time polymerase chain reaction.

**Gene**	**Primer sequences**	
	**Forward**	**Reverse**
Glyceraldehyde 3-phosphate dehydrogenase (*Gapdh*)	5′–TGTGTCCGTCGTGGATCTGA–3′	5′–TTGCTGTTGAAGTCGCAGGAG–3′
Tenascin C (*Tnc*)	5′– GGAGCAAGCTGATCCAAACCA –3′	5′-CCAGTGCTTGAGTCTTGTCACCA-3′
Fibronectin (*Fn*)	5′–GTGGTCATTTCAGATGCGATTCA–3′	5′–ATTCCCGAGGCATGTGCAG–3′
Periostin (*Postn*)	5′–CAGTTGGAAATGATCAGCTCTTGG–3′	5′–CAATTTGGATCTTCGTCATTGCAG–3′
Type I collagen (*Col1*)	5′–GGGTCCCTCGACTCCTACA–3′	5′–TGTGTGCGATGACGTGCAAT–3′
Runt-related transcription factor-2 (*Runx2*)	5′–GCCCAGGCGTATTTCAGA–3′	5′–TGCCTGGCTCTTCTTACTGAG–3′
Osterix (*Osx*)	5′–GAAAGGAGGCACAAAGAAG–3′	5′–CACCAAGGAGTAGGTGTGTT–3′
Alkaline phosphatase (*Alp*)	5′–ATCTTTGGTCTGGCTCCCATG–3′	5′–TTTCCCGTTCACCGTCCAC–3′

### Cell Cultures

Mouse embryonic palatal mesenchymal (MEPM) cells were isolated from secondary palatal shelves dissected from ICR mouse embryos at E13.5–14.0. The MEPM cells were a mixture of cells obtained from the anterior and posterior portions of the palatal shelves. MEPM cells were cultured in Dulbecco’s Modified Eagle Medium/Nutrient Mixture F-12 (DMEM/F12; Gibco, Thermo Fisher Scientific) containing 10% fetal bovine serum (FBS; Gibco, Thermo Fisher Scientific) and 1% penicillin-streptomycin (Roche Diagnostics, Berlin, Germany). For stability, the medium was changed to FBS-free DMEM (Gibco, Thermo Fisher Scientific) containing 50 ng/mL recombinant human TGF-β3 (PeproTech, London, United Kingdom) or 100 ng/mL SHH (R&D Systems) for 0, 24, or 48 h. In some experiments, the cells were pre-treated with 10 μM SB203580 (a p38 inhibitor; Adipogen Life Sciences, San Diego, CA, United States) or 1.0 μM SIS3 (a Smad3 inhibitor; Cayman Chemical Company, Ann Arbor, MI, United States) for 24 h prior to the addition of TGF-β3.

O9-1 cells (Merck Millipore) were derived from mass cultures of Wnt1-Cre/R26R-GFP reporter-expressing cranial neural crest cells from E8.5 mouse embryos. O9-1 cells were seeded on a dish coated with Matrigel^TM^ (Thermo Fisher Scientific) and cultured in Complete ES Cell Medium containing 15% fetal bovine serum, mouse leukemia inhibitory factor (mLIF; Merck Millipore) and 25 ng/mL recombinant human basic fibroblast growth factor (rhbFGF; Kaken Pharmaceutical Co., Ltd, Tokyo, Japan). For stability, the medium was changed to FBS-free Minimum Essential Medium-α (Wako Pure Chemical Corporation, Osaka, Japan) containing 10 ng/mL recombinant human TGF-β3 (PeproTech) or 100 ng/mL SHH (R&D Systems) for 0, 24, or 48 h.

### Enzyme-Linked Immunosorbent Assay (ELISA)

The concentration of TNC in the medium bathing cultured cells was measured using an ELISA kit (27767; IBL, Fujioka, Japan). Dilute samples of culture medium were added to 96-well plates and incubated for 1 h at 37°C. Horseradish peroxidase-conjugated mouse anti-TNC monoclonal antibody (clone 4F10TT, IBL, Fujioka, Japan) was added to each well and incubated for 30 min at 4°C. Substrate solution was then added to each well, and the mixture was incubated for 30 min at room temperature. Subsequently, stop solution was added to each well, and the level of TNC was determined with a microplate reader at 450 nm (ImmunoMini NJ-2300; NJ InterMed, Tokyo, Japan).

### Statistical Analysis

Data are presented as the mean ± standard deviation. Comparisons between groups were made using the Mann–Whitney *U* test. A *P*-value < 0.05 was considered significant.

## Results

### TNC Is Expressed More Strongly in the Posterior Palatal Shelves Than Tenascin-W (TNW) or Tenascin-X (TNX) During Palatal Development

**FIGURE 1:**
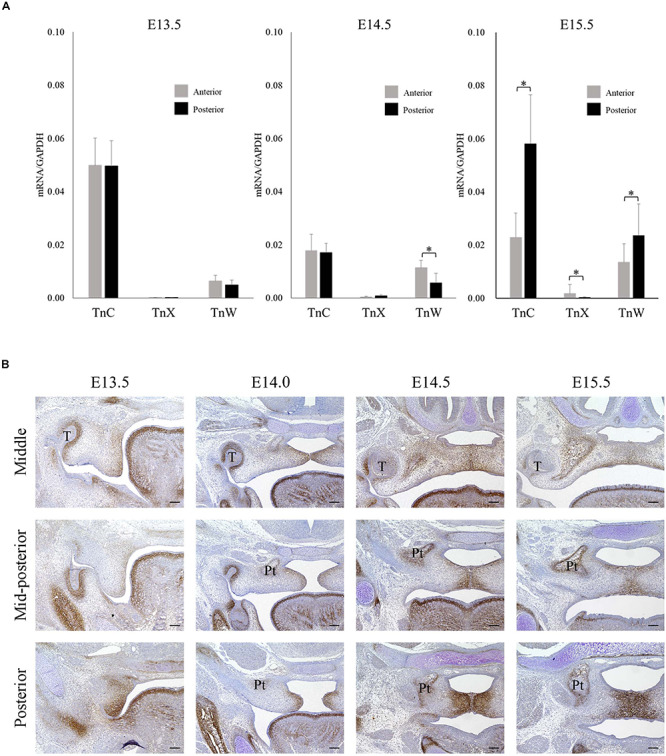
Tenascin C expression during palatal development in mice between embryonic day 13.5 (E13.5) and E15.5. **(A)** The mRNA expressions of tenascin-C (TNC), tenascin-X (TNX), and tenascin-W (TNW) in the anterior and posterior palatal shelves at E13.5, E14.5, and E15.5 (real-time polymerase chain reaction). The mRNA expression of TNC was greater than that of TNX or TNW and significantly higher in the posterior palate than in the anterior palate at E15.5. Data are presented as the mean ± standard deviation (*n* = 6). The mRNA expressions were normalized to that of glyceraldehyde 3-phosphate dehydrogenase (GAPDH). ^∗^*P* < 0.05. **(B)** Immunofluorescence staining of TNC protein in the middle, mid-posterior and posterior regions of the palatal shelves at E13.5, E14.0, E14.5, and E15.5. Stronger TnC expression was observed in the posterior palatal shelves than in the middle or mid-posterior regions, particularly at the later developmental stages. T, tooth germ; Pt, pterygoid process. Bar = 100 μm.

Real-time PCR was used to investigate the mRNA expressions of TNC, TNX, and TNW in the anterior and posterior palatal shelves at three time points (E13.5, E14.5, and E15.5) during palatal development. The mRNA expression of TNC in the palatal shelves was greater than that of TNX or TNW (Figure 1A). Furthermore, TNC mRNA expression was significantly higher in the posterior palate than in the anterior palate at E15.5 (Figure 1A). Immunohistochemistry experiments were performed to examine the expression of TNC protein in the posterior palate. TNC was widely expressed in the palatal mesenchyme and underneath the palatal epithelium (Figure 1B). Evaluation of the immunostaining intensity along the anterior–posterior axis of the palatal shelves revealed a higher level of TNC expression in the posterior portion than in the middle or mid-posterior regions, and this trend was particularly evident at the later developmental stages of palatogenesis (E14.5 and E15.5; Figure 1B). Therefore, the immunohistologic observations support the results of the real-time PCR experiments.

### TGF-β3 and SHH Induce TNC Expression in MEPM Cells *in vitro*

**FIGURE 2:**
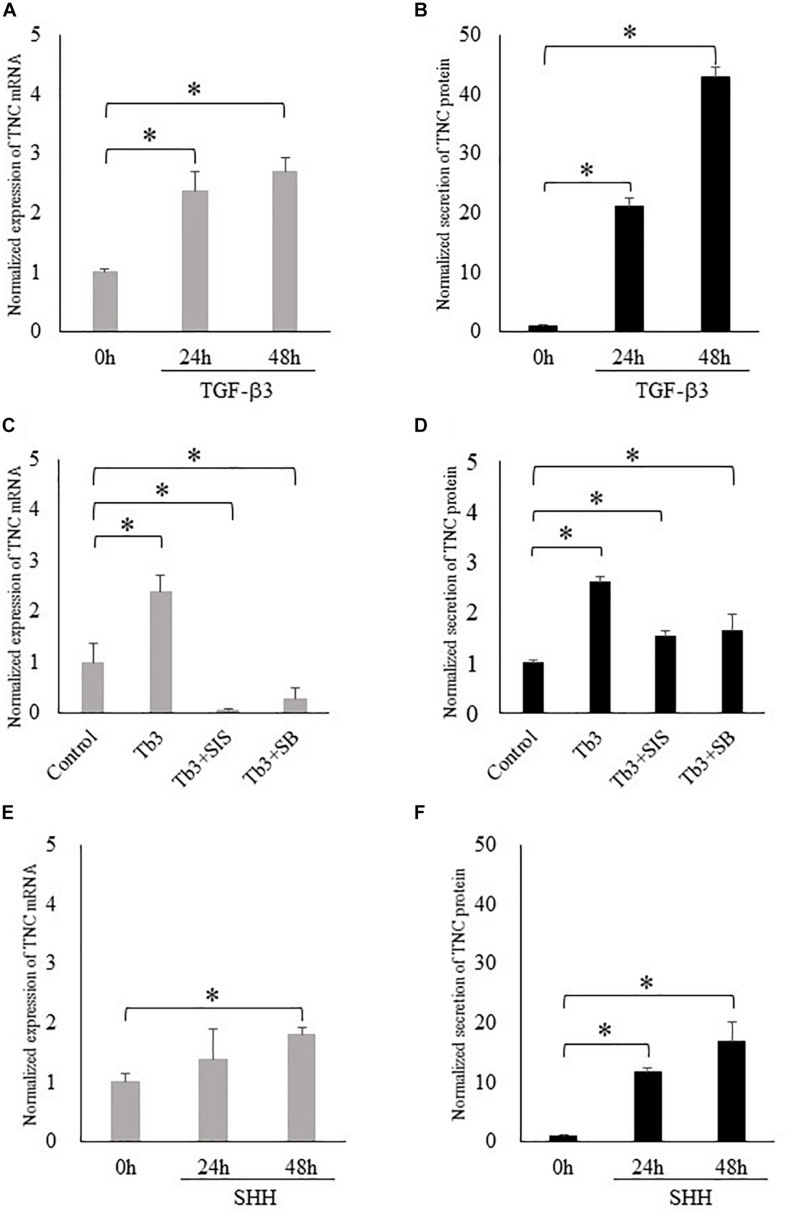
Transforming growth factor-beta-3 (TGF-β3) and sonic hedgehog (SHH) increase the expression of tenascin-C (TNC) in mouse embryonic palatal mesenchymal (MEPM) cells (embryonic day 13.5–14.0). **(A,C,E)** The mRNA expression of tenascin-C (TNC) measured using real-time polymerase chain reaction. **(B,D,F)** The amount of TNC protein secreted into the culture medium measured by enzyme-linked immunosorbent assay. TGF-β3 increased the expression of TNC mRNA **(A)** and the secretion of TNC protein **(B)** by MEPM cells at 24 h and 48 h. **(C,D)** These effects of TGF-β3 (Tb3) were inhibited by a Smad3 inhibitor (Tb3 + SIS) and a p38 inhibitor (Tb3 + SB). SHH also enhanced the expression of TNC mRNA **(E)** and the secretion of TNC protein **(F)** by MEPM cells. Data are expressed as the mean ± standard deviation (*n* = 4) and were normalized to the 0 h or control values. ^∗^*P* < 0.05.

Based on our observation of strong TNC expression in the posterior palatal shelves, we speculated that TNC may play an important role in the development of the soft palate. Since TGF-β3 mutations are associated with cleft palate and bifid uvula in humans (Bertoli-Avella et al., 2015), we hypothesized that TGF-β regulates the expression of TNC in palatal mesenchyme. In cultured MEPM cells, TGF-β3 increased the expression of TNC mRNA and the secretion of TNC protein at 24 h and 48 h (Figures 2A,B). These effects of TGF-β3 were prevented by inhibitors of Smad3 or p38, which are known mediators of TGF-β signaling during palatal development (Figures 2C,D) (Iwata et al., 2011). Since it has been reported that the expression of SHH in palatal epithelium is diminished in mice deficient in TGF-β3 (Sasaki et al., 2007; Ozturk et al., 2013), we also hypothesized that SHH regulates the expression of TNC mRNA in palatal mesenchyme. SHH also enhanced the expression of TNC mRNA and secretion of TNC protein by MEPM cells at 48 h (Figures 2E,F).

### Loss of TGF-β Signaling in the Palatal Epithelium Reduces TNC Expression in the Palatal Mesenchyme

**FIGURE 3:**
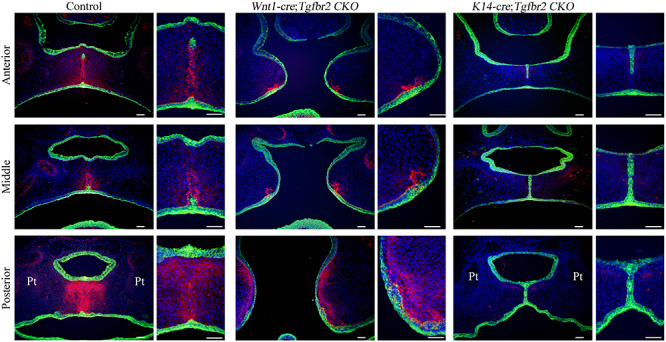
Disruption of transforming growth factor-beta (TGF-β) signaling in the palatal epithelium reduces tenascin-C (TNC) expression in the palatal mesenchyme. The images show TNC (pink) and K14 (green) expression during palatal development in control mice, *Wnt1-cre*;*Tgfbr2*^*fl/fl*^ mice (which lack the TGF-β type II receptor in palatal mesenchymal cells) and *K14-cre*;*Tgfbr2*^*fl/fl*^ mice (which lack the TGF-β type II receptor in palatal epithelial cells) at embryonic day 15.5. Each narrow image is an enlarged view of part of the wider image to its left. Cell nuclei were stained blue with 4′,6-diamidino-2-phenylindole (DAPI). Pt, pterygoid process. Bar = 50 μm.

Since the above findings support the possibility that TNC expression is regulated by TGF-β signaling and plays an important role in soft palate development, we performed immunohistochemistry experiments to evaluate TNC expression in *Tgfbr2* conditional knockout mice that exhibit cleft palate. Interestingly, TNC expression was dramatically reduced in *K14-cre*;*Tgfbr2*^*fl/fl*^ mice that lack the TGF-β type II receptor in palatal epithelial cells but not decreased in *Wnt1-cre*;*Tgfbr2*^*fl/fl*^ mice that lack the TGF-β type II receptor in palatal mesenchymal cells (Figure 3). To facilitate orientation, Supplementary Figure S1 presents lower-magnification images of HE-stained sections adjacent to those shown in Figure 3. *K14-cre*; *Tgfbr2*^*fl/fl*^ mice exhibit a cleft soft palate caused by a defect in the disappearance of the medial edge epithelium (Xu et al., 2006). These observations indicate that TGF-β signaling in the palatal epithelium plays an important role in the paracrine regulation of TNC expression in palatal mesenchyme. We also confirmed that *Tgfb3* mutant mice, which exhibit cleft palate because of a loss of TGF-β3 in the palatal epithelium, also lacked TNC expression in the palatal mesenchyme (data not shown).

### The Effect of TGF-β and SHH in a Cranial Neural Crest Cell Line

**FIGURE 4:**
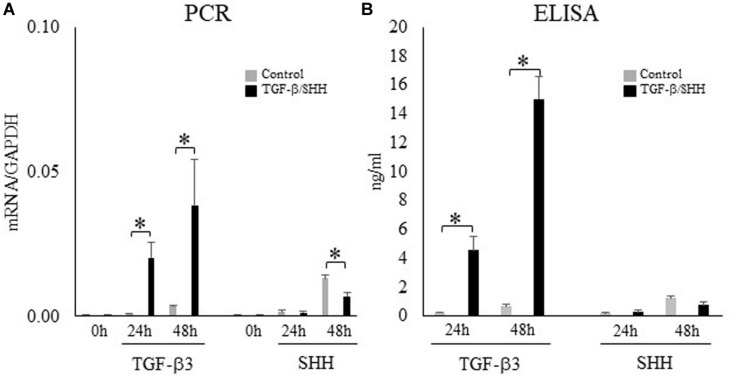
Transforming growth factor-beta-3 (TGF-β3) but not sonic hedgehog (SHH) stimulates the expression of tenascin-C (TNC) mRNA and protein in O9-1 cells. **(A)** The mRNA expression of TNC in O9-1 cells cultured with or without TGF-β3 or SHH for 0 h, 24 h or 48 h (real-time polymerase chain reaction). The mRNA expressions were normalized to that of glyceraldehyde 3-phosphate dehydrogenase (GAPDH). **(B)** Secretion of TNC protein by O9-1 cells cultured with or without TGF-β3 or SHH for 24 h or 48 h (enzyme-linked immunosorbent assay). Data are expressed as the mean ± standard deviation (*n* = 4). ^∗^*P* < 0.05.

During palatogenesis, TGF-β3 is expressed continuously in the medial edge epithelium along the entire anterior–posterior axis of the palate. By contrast, SHH does not show continuous expression; instead, SHH is found in the palatal rugae of the anterior hard palate during early palatal development and then expressed at a later stage in the posterior soft palate (Welsh and O’Brien, 2009). The differences in the spatiotemporal patterns of TGF-β3 and SHH expression in the palatal epithelium cannot explain the characteristic expression of TNC in the posterior region. Therefore, we investigated the functional significance of TGF-β and SHH signaling to palatal mesenchymal cells during palatogenesis, not only in terms of TNC expression but also with regard to cell differentiation or fate. O9-1 cells (a cranial neural crest cell line) were first cultured with medium containing mLIF and rhbFGF to prevent progression of differentiation and then challenged with TGF-β3 or SHH. TGF-β3 was found to induce the expression of TNC mRNA in O9-1 cells (real-time PCR; Figure 4A) and enhance the secretion of TNC protein by these cells (ELISA; Figure 4B). Surprisingly, SHH did not stimulate TNC mRNA expression (Figure 4A) or TNC protein secretion (Figure 4B) in O9-1 cells.

### TGF-β Signaling Promotes Fibroblastic Differentiation and Inhibits Osteoblastic Differentiation of Cranial Neural Crest Cells

**FIGURE 5:**
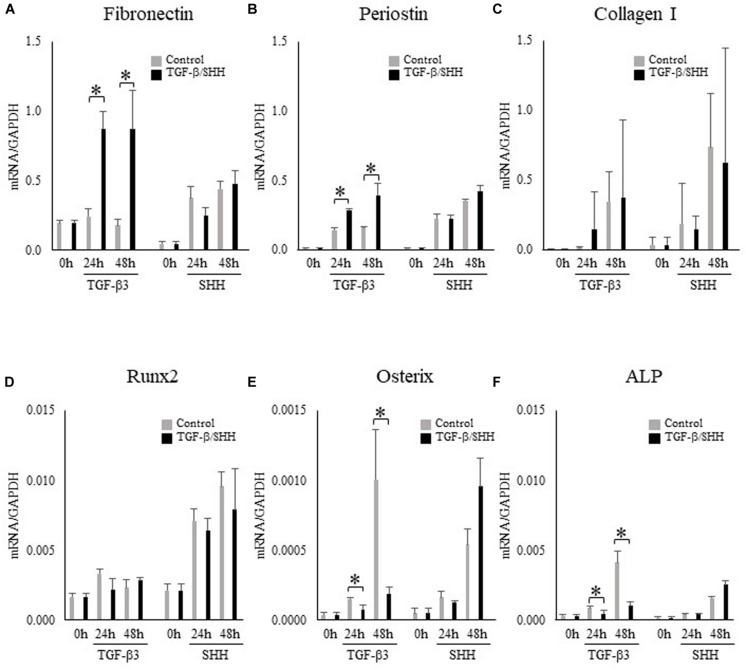
Effects of transforming growth factor-beta-3 (TGF-β3) and sonic hedgehog (SHH) on the expression of fibroblastic and osteoblastic cell markers in O9-1 cells. **(A–C)** The mRNA expressions of the fibroblastic cell markers, fibronectin **(A)**, periostin **(B)** and type I collagen **(C)** in O9-1 cells. **(D–F)** The mRNA expressions of the osteoblastic cell markers Runx2 **(D)**, osterix **(E)** and alkaline phosphatase (ALP) **(F)** in O9-1 cells. The mRNA expressions were normalized to that of glyceraldehyde 3-phosphate dehydrogenase (GAPDH). Data are expressed as the mean ± standard deviation (*n* = 4). ^∗^*P* < 0.05.

Because SHH was able to induce TNC expression in MEPM cells but not O9-1 cells, we hypothesized that the regulation of TNC expression by SHH signaling might depend on cell type. Visual inspection of the O9-1 cells revealed a morphologic change to a fibroblastic appearance after stimulation for 24 h with TGF-β3, whereas no obvious alteration in cell shape was observed after challenge with SHH. Real-time PCR demonstrated that O9-1 cells showed upregulation of fibroblastic markers, including fibronectin (Figure 5A) and periostin (Figure 5B), and downregulation of osteogenic markers, including osterix (Figure 5E) and alkaline phosphatase (Figure 5F), after stimulation with TGF-β3 for 24 h or 48 h. Interestingly, administration of SHH for 24 h or 48 h was without effect on fibroblastic and osteogenic marker expression (Figure 5). The above findings suggest that TGF-β3 can induce O9-1 cells to differentiate into fibroblastic cells, whereas SHH does not affect the differentiation of immature mesenchymal cells in the palatal shelves.

### Loss of SHH Signaling in the Posterior Region of the Palatal Epithelium Leads to Decreased TNC Expression in the Posterior Palatal Mesenchyme

**FIGURE 6:**
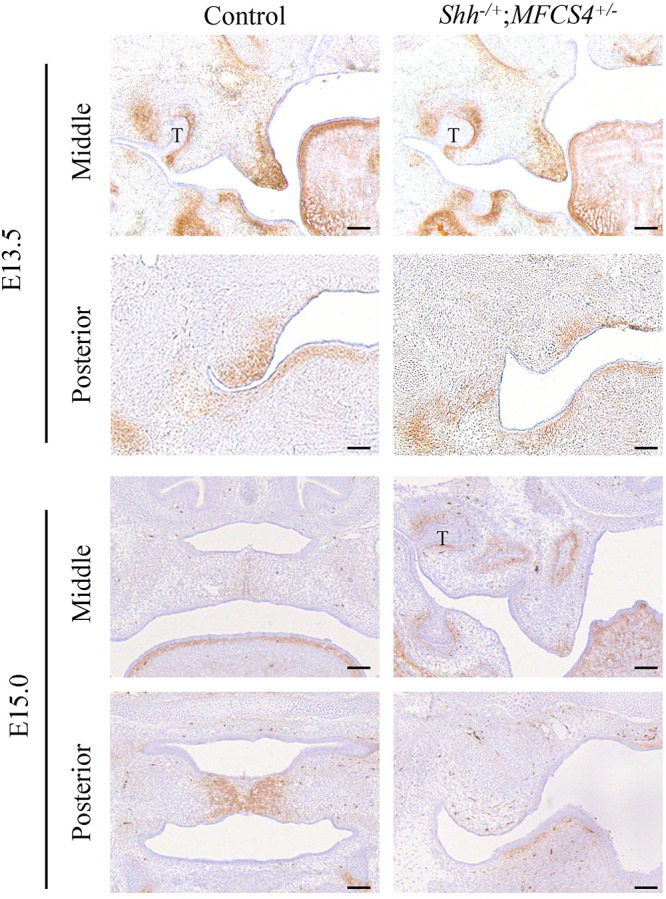
Tenascin-C (TNC) expression during palatal development in *Shh^–/+^*;*MFCS4*^+/–^ mice. The expression of TNC in the anterior and posterior palatal shelves at embryonic day 13.5 (E13.5) and E15.0 was evaluated using immunohistochemistry techniques. T, tooth germ. Bar = 100 μm.

In view of the differing responses of O9-1 cells to TGF-β3 and SHH, we compared TNC expression in the posterior region of the palatal mesenchyme between SHH-deficient mice and wild-type mice. SHH expression in the palatal epithelium is restricted to the area of the rugae (Welsh and O’Brien, 2009; Xu et al., 2016). Remarkably, SHH expression in the posterior palatal epithelium is delayed compared with the middle portion because of restricted regulation by the SHH enhancer, MFCS4, in the posterior part of the palate and oropharyngeal region (Sagai et al., 2009). Therefore, we prepared compound heterozygous mice (*Shh^–/+^*;*MFCS4*^+/–^) in which SHH was only deleted in the posterior palatal shelves. Immunohistochemistry experiments demonstrated that TNC expression in the posterior region of the palatal shelf of *Shh^–/+^*;*MFCS4*^+/–^ mice was slightly reduced at E13.5 and completely lost at E15.0 (Figure 6). By contrast, TNC expression was detected in the anterior region of the palatal shelf of *Shh^–/+^*;*MFCS4*^+/–^ mice at both E13.5 and E15.0 (Figure 6). These results indicate that TNC expression in the posterior palatal mesenchyme is regulated by SHH signaling.

## Discussion

Recent progress in palatogenesis research has provided important insights into the genes responsible for cleft palate, and there has been particular interest in the role of spatiotemporal signaling between the anterior and posterior axis (Li et al., 2017). The NIH FaceBase consortium has produced a comprehensive review of the transcription factors, growth factors and receptors that are specifically expressed in the palatal region (Potter and Potter, 2015). Nevertheless, there are various types of cleft palate, and the mechanisms underlying submucous cleft palate and partial cleft palate remain incompletely understood.

Mutations of the genes encoding TGF-β ligands, Smads and their receptors have been reported as candidate mechanisms for cleft soft palate or bifid uvula in humans (Bertoli-Avella et al., 2015). Although TGF-β signaling is recognized to have particular significance in the posterior palatal mesenchyme, TGF-β3 is expressed continuously throughout the entire palatal epithelium (Taya et al., 1999). Furthermore, we have reported that Smad2 and Smad3, which are transcriptional factors involved in TGF-β signaling, are also activated in the palatal epithelium throughout the entire anterior–posterior axis (Higa et al., 2016). However, the phenotype of mice with conditional knockout of the TGF-β type I receptor (*K14-cre*;*Tgfbr1*^*fl/fl*^) and TGF-β type II receptor (*K14-cre*;*Tgfbr2*^*fl/fl*^) in the palatal epithelium is partial cleft of the posterior palatal shelves, with no developmental abnormality of the anterior portion. This apparent discrepancy raises the possibility that signaling between the palatal epithelium and mesenchyme varies along the anterior–posterior axis. In palatal mesenchyme, many growth factors and transcriptional factors show differential expression along the anterior–posterior axis, and this is considered important for the development of the palate (Smith et al., 2012). However, the role of ECM proteins along the anterior–posterior axis has not been characterized previously. TGF-β is known as an important regulator of ECM production. Therefore, we investigated how the expression pattern of an ECM component (TNC) was influenced by TGF-β signaling and how this was related to cell fate, which is relevant to the formation of bone in the anterior palate and connective tissue in the posterior palate.

TNC confers ‘anti-adhesive’ properties to the ECM and plays an important role in embryonic morphogenesis and tissue repair during wound healing. TNC exhibits characteristic expression in the posterior end of the palatal shelves during palatogenesis. In the present study, we demonstrated that TGF-β3 upregulated TNC expression in primary MEPM cells and O9-1 cells, both of which are derived from the cranial neural crest. It has been reported that a binding element in the proximal region of the TNC promoter mediates the responsiveness to TGF-β, which involves Smad3/4, Sp1, Ets1, and CBP/p300 (Jinnin et al., 2004). The present study investigated the direct transcriptional regulation of TNC expression by TGF-β. However, somewhat unexpectedly, we found that TNC expression was dramatically reduced in *K14-cre*;*Tgfbr2*^*fl/fl*^ mice (which lack the TGF-β type II receptor in palatal epithelial cells) but not decreased in *Wnt1-cre*;*Tgfbr2*^*fl/fl*^ mice (which lack the TGF-β type II receptor in palatal mesenchymal cells). Iwata et al. (2012) showed that loss of the TGF-β type II receptor in the palatal mesenchyme led to upregulation of the expression of TGF-β2 and acceleration of a non-canonical TGF-β signaling pathway via a TGF-β type I/III receptor complex. We speculate that TNC expression in *Wnt1-cre*;*Tgfbr2*^*fl/fl*^ mice may have been maintained by this non-canonical TGF-β signaling pathway. Since *K14-cre*;*Tgfbr2*^*fl/fl*^ mice exhibit partial cleft of the posterior palate, this suggests that the development of the anterior hard palatal mesenchyme is not affected by the loss of TNC expression caused by defective TGF-β signaling in the palatal epithelium.

Paracrine regulation of TNC expression in the posterior palatal mesenchyme by TGF-β in the posterior palatal epithelium requires the secretion of a TGF-β-regulated factor from the epithelium that targets genes in the mesenchyme. Therefore, we explored the possible role of SHH signaling in palatal epithelium (Welsh and O’Brien, 2009; Lee et al., 2011), since the expression of SHH in palatal rugae is diminished in mice deficient in TGF-β3 (Sasaki et al., 2007; Ozturk et al., 2013). Interestingly, we found that SHH induced TNC expression in primary cells from embryonic palatal mesenchyme (MEPM cells) but not in O9-1 cells, which are undifferentiated cranial neural crest cells. Since the primary MEPM cells were harvested on E13.5–E14.0, they would have been a complex population of cells such as osteogenic and fibrogenic progenitors. We speculate that SHH induces TNC expression in fibrogenic cells but not immature cells. This would explain the lack of effect of SHH on TNC expression in O9-1 cells, which were maintained as immature cells before stimulation with SHH. Furthermore, SHH had no effect on the expression of osteoblastic and fibroblastic markers in O9-1 cells. Based on the above observations, we suggest that SHH does not influence the determination of cell fate during the early embryonic developmental stage but can do so in palatal mesenchyme at a later developmental stage. The above findings are also consistent with the lack of TNC expression in *Shh^–/+^*;*MFCS4*^+/–^ mice, which have delayed expression of SHH in the posterior region of the palatal shelves as compared with the anterior and middle regions (Sagai et al., 2009). We generated these compound heterozygous mice to avoid early embryonic lethality, utilizing the fact that MFCS4, an enhancer of SHH, is expressed only in the region of the soft palate. Interestingly, homozygous MFCS4 mutant mice have soft palates that are shortened in length (Sagai et al., 2009), indicating that deficiency of SHH in the posterior palatal epithelium impairs the growth of the soft palate but does not affect palatal fusion. The regulation of TNC expression by SHH signaling in the posterior soft portion during the later stages of palatogenesis may also affect the development of the palatal muscles via actions on myofibrogenic cells. More recently, Li et al. (2018) reported that *K14-cre*;*R26SmoM2* mice with constitutive activation of SHH signaling in the palatal epithelium exhibit a submucous cleft palate. The authors suggested that constitutive activation of hedgehog signaling resulted in dysfunction of the p63/Irf6 regulatory loop that is necessary for the disappearance of the medial edge epithelium during fusion of the palatal shelves. However, the study of Li et al. (2018) did not consider the role of SHH signaling in palatal mesenchyme.

The Foxf2 transcriptional factor is a downstream target of SHH expression in palatal epithelium, which in turn is regulated by FGF-10 expressed by palatal mesenchyme. Furthermore, *Foxf2^–/–^* mice show downregulated expression of TGF-β ligands and TNC as well as reduced phosphorylation of Smad2/3 (Nik et al., 2016). We have also confirmed that TGF-β3 is expressed in the posterior palatal epithelium in *Shh^–/+^*;*MFCS4*^+/–^ mice using *in situ* hybridization experiments (data not shown). We initially considered that SHH may be a downstream target of TGF-β3 in palatal epithelium for regulation of TNC expression. However, we found that *pCag-CreERTM*;*Shh*^*fl/fl*^ mice exhibit decreased TGF-β3 expression in skeletal muscle and reduced phosphorylation of Smad2/3 (Okuhara et al., 2019). This raises the possibility that both TGF-β3 and SHH act as major mediators of epithelial–mesenchymal cross-talk during palatogenesis, with this signaling loop regulating proliferation, migration and ECM deposition in the palatal mesenchyme. Even partial collapse of this signaling loop would impair normal palatogenesis, and the regulation of this loop may be more sensitive to disturbance in the posterior palatal epithelium.

Soft palate development is thought to involve many different growth factors including Wnt and FGF (Janeckova et al., 2019). It has been reported that Wnt-β-catenin signaling is disrupted in *Tgfbr2*^*fl/fl*^;*K14-Cre* mice by the upregulated expression of Dickkopf-related protein-1 (Dkk1) and Dkk4 (Iwata et al., 2014) and that Wnt5a induces the expression of TNC in osteogenic cells (Morgan et al., 2011). Based on the above reports, it is possible that osteoblastic and fibroblastic cell differentiation may involve competition between Wnt and TGF signaling through TNC expression.

On the other hand, Rice et al. (2014) have shown that FGF-10 is expressed in the palatal mesenchyme and that exogenous FGF-10 induces SHH expression in the palatal epithelium in wild-type but not *Fgfr2b*^–/–^ mutant mice. Furthermore, *Fgf10^–/–^* and *Fgfr2b^–/–^* mutant mice show altered patterns of TGF-β3 and SHH expression in the palatal epithelium (Alappat et al., 2005; Rice et al., 2014). Since FGF receptor-2b is expressed in palatal epithelium, epithelial–mesenchymal interactions may involve FGF-10–SHH–TNC signaling in addition to the TGF-β3–SHH–TNC pathway described in the present study.

TNC functions as an adhesion-modulating ECM protein that inhibits the adhesive effects of fibronectin (Chiquet-Ehrismann and Chiquet, 2003). Fibronectin is expressed ubiquitously in palatal mesenchyme (Tang et al., 2015). The orientation/alignment of fibronectin fibers during palatal development is important for morphologic changes in the palatal shelves such as the elevation process. The anti-adhesive function of TNC in fibronectin-expressing regions might play a role in the regulation of cell migration or aggregation that contributes to the changes in palatal shelf morphology. The initial studies of TNC-knockout mice did not report any obvious developmental abnormalities such as cleft palate (Saga et al., 1992; Forsberg et al., 1996), suggesting the existence of mechanisms that compensate for the loss of TNC. In the present study, we also detected TNW expression in the palatal shelves (Figure 1A). Therefore, TNW might be one candidate that compensates for a lack of TNC during palatal development. Furthermore, we also speculate that the orientation of the palatal muscles might be disrupted in TNC-knockout mice. Additionally, TNC function is required for recovery from pathologic events such as wounds, fibrosis or inflammation (Tamaoki et al., 2005). In future, TNC may be an important biomaterial for use in reconstruction of the soft palate using regenerative medicine techniques. Further studies are needed to clarify the role of TNC in posterior palatogenesis.

## Data Availability Statement

The datasets generated for this study are available on request to the corresponding author.

## Ethics Statement

The animal study was reviewed and approved by Animal Experiment Committee of Fukuoka Dental College, Fukuoka, Japan (nos. 17012 and 18005).

## Author Contributions

KOk contributed to the conception and design of the study SOh, KOg, SOk, MT-N, ST, and MR contributed to data acquisition. MO, SI, and TS contributed to the analysis and interpretation of the results. KOk contributed to the writing of the manuscript. All authors read and approved the submitted version.

## Conflict of Interest

The authors declare that the research was conducted in the absence of any commercial or financial relationships that could be construed as a potential conflict of interest.

## Footnotes

1 https://www.facebase.org/mouseanatomy/
