# Supplementary figures and images for: Transforming Growth Factor-Beta and Sonic Hedgehog Signaling in Palatal Epithelium Regulate Tenascin-C Expression in Palatal Mesenchyme During Soft Palate Development
Source: Front Physiol. 2020 Jun 4;11:532. doi: 10.3389/fphys.2020.00532 (PMC7287209; doi:10.3389/fphys.2020.00532)

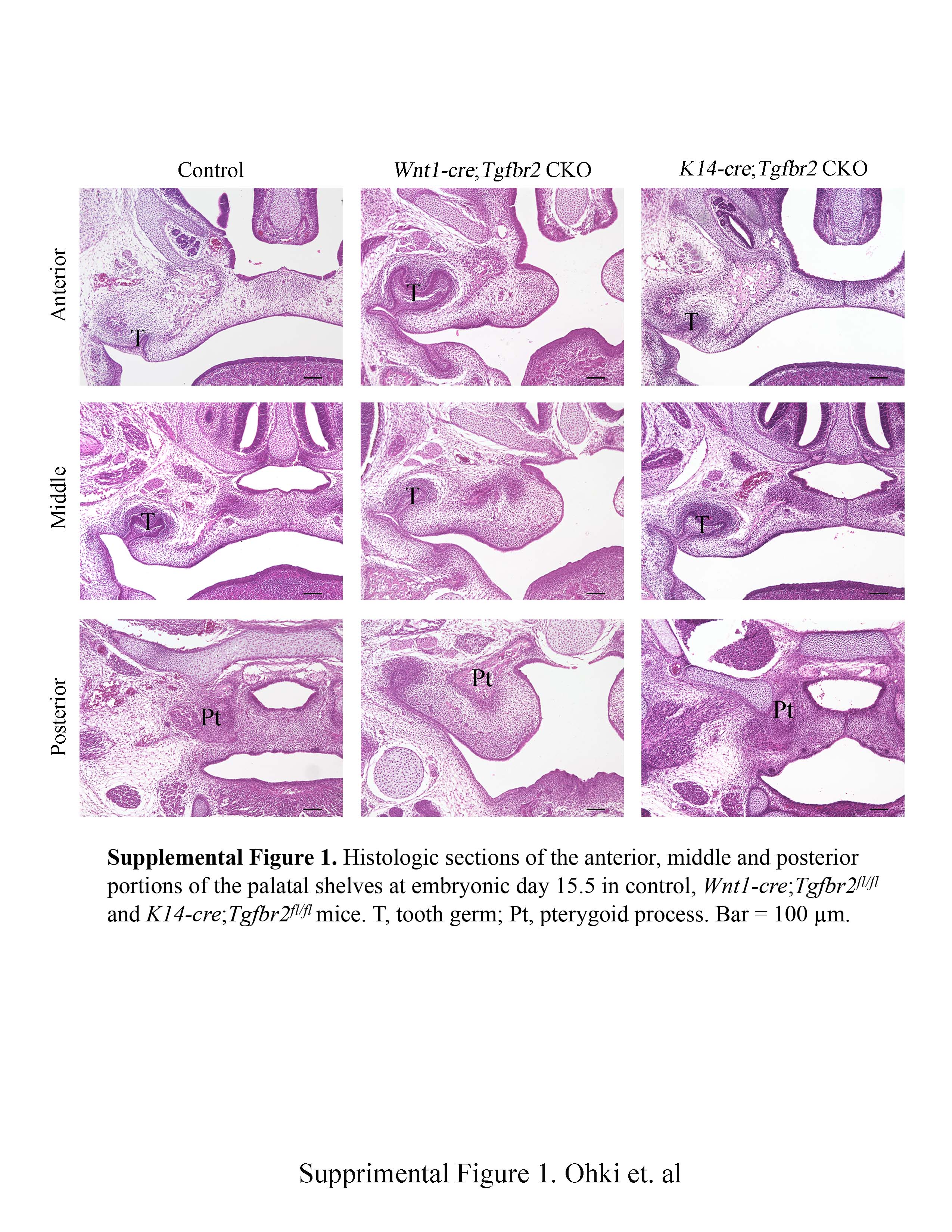

Supplement: Supplementary file 1 [file Image_1.jpg]
